# Outcomes of Abductor Repair Using Mesh Augmentation in Oncologic Proximal Femur Replacement

**DOI:** 10.3390/curroncol31100425

**Published:** 2024-09-24

**Authors:** Samuel E. Broida, Harold I. Salmons, Aaron R. Owen, Matthew T. Houdek

**Affiliations:** Department of Orthopedic, Mayo Clinic, Rochester, MN 55905, USA; broida.samuel@mayo.edu (S.E.B.); salmons.harold@mayo.edu (H.I.S.); owen.aaron@mayo.edu (A.R.O.)

**Keywords:** endoprosthesis, abductor repair, mesh, proximal femur, metastatic disease

## Abstract

Reconstruction of the abductor mechanism remains a primary challenge with contemporary proximal femoral replacement (PFR) surgery. Previously, techniques such as trochanteric preservation or direct repair to the implant have been described; however, these strategies are limited in their ability to tension the repair and reattach other muscles of the hip girdle. The aim of this study was to evaluate the outcomes of patients undergoing oncologic PFR using a novel technique of mesh augmentation for soft tissue repair. Methods: We reviewed 18 (mean age 64 years; 8 female: 10 male) consecutive patients undergoing PFR with Marlex mesh augmentation between 2018 and 2023 at a single institution. The most common indication was metastatic disease (n = 13). The mean follow-up in the 14 surviving patients was 27 months (range 12–34). Results: All patients were ambulatory at final follow-up. There were no post-operative dislocations, infections, or wound issues. At the final follow-up, the mean total MSTS score was 77%. Conclusion: Mesh augmentation of PFRs allowed for adequate soft tissue tensioning and muscular attachment to the body of the implant. In our series, this technique was durable, with no dislocations and no mesh-related complications. In summary, mesh augmentation of PFRs may be considered during reconstruction for oncologic indications.

## 1. Introduction

To achieve sufficient margins, oncologic proximal femur resection often sacrifices important attachment sites for muscles, which aid in hip function and stability. Endoprosthetic reconstruction via proximal femoral replacement (PFR) allows for early weight-bearing; however, instability related to soft tissue deficiency is the most common mode of failure [1,2,3]. The inability to achieve biological fixation at the tendon–prosthetic interface has led to the development of numerous techniques to attempt to restore muscular attachments to the prosthesis [4,5,6,7,8,9].

The two primary modes of soft-tissue reconstruction include (1) soft-tissue-only repair, whereby the abductors are sutured directly to the body of the implant or local soft tissues, and (2) bone repair, in which the greater trochanter is preserved when possible and advanced to the prosthesis [4,5,6,7]. Other proposed techniques include augmenting soft-tissue-only repair with products such as Aortograft [8] or a Trevira tube [9], which act as a sleeve for soft tissue attachments. However, these products are not readily available in most settings, are difficult to secure to the body of the prosthesis, and do not allow for tissue ingrowth.

At our institution, a novel technique using an off-the-shelf sheet of Marlex mesh (Bard, Franklin Lakes, NJ, USA) is used to envelop the proximal femoral prosthesis and serve as a circumferential attachment site for the hip musculature. Given the known tendency of Marlex mesh to induce an inflammatory and fibroblastic local tissue reaction [10,11,12], scar formation at the mesh-tendon interface may provide a stronger and more durable repair than other contemporary techniques.

As this technique has not been previously described, we sought to evaluate the outcomes of patients undergoing PFR with Marlex mesh to augment the soft tissue-only repair. The primary purpose of this study was to evaluate ambulatory status, gait aid utilization, and the presence of a Trendelenburg limp. The secondary purpose was to evaluate clinical outcomes and complications following this procedure. It is the authors’ hypothesis that this technique allows for good functional outcomes with a low rate of dislocations and mesh-related complications.

## 2. Materials and Methods

Patients who underwent oncologic proximal femoral replacement at a single academic institution from 1 January 2020 to 1 August 2023 were identified. Patients whose reconstruction included a soft tissue-only repair with mesh augmentation were included. Clinical documentation, operative notes, pathology reports, and radiologic images were screened for inclusion. Patients who underwent total femur replacement or had less than six months of follow-up were excluded. This study was initiated after approval from the institutional review board.

Eighteen patients met the criteria for inclusion (Table 1). The mean age at the time of surgery was 64 years (range 37–78), the mean body mass index (BMI) was 30 kg/m^2^ (range 20–42), and 44% of patients were female. Four patients underwent PFR for treatment of a primary sarcoma: chondrosarcoma, osteosarcoma, undifferentiated pleomorphic sarcoma, and mast cell sarcoma. Thirteen patients underwent PFR for resection of metastatic disease consisting of renal cell carcinoma (n = 9), lung adenocarcinoma (n = 1), prostate adenocarcinoma (n = 1), breast adenocarcinoma (n = 1), and multiple myeloma (n = 1). One patient with a remote history of undifferentiated pleomorphic sarcoma underwent PFR for a radiation-associated fracture eight years after radiation therapy. Metastatic lesions were located in the subtrochanteric region (n = 5), intertrochanteric region (n = 7), and femoral neck (n = 1). Six patients had a history of radiotherapy to the proximal thigh (mean 4375 cGy), and seven patients received adjuvant radiation (mean 2400 cGy) following PFR. Four patients underwent neoadjuvant chemotherapy for primary sarcoma.

### 2.1. Technique

**Figure 1:**
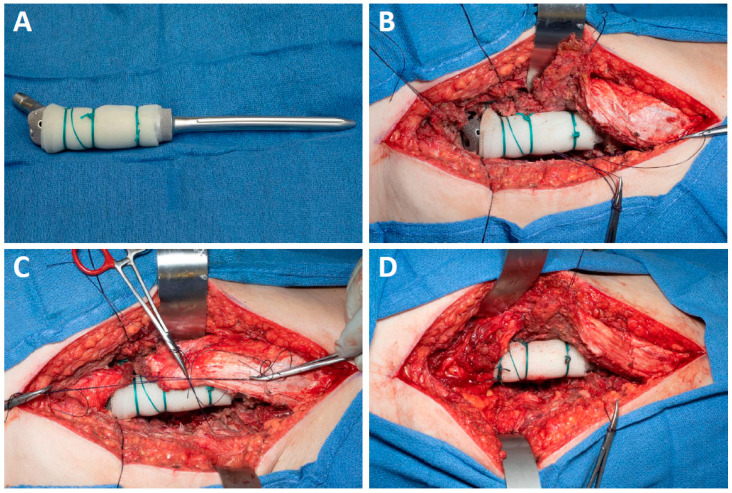
(**A**) Marlex mesh is secured to the body of the prosthesis; (**B**) Abductors, external rotators, gluteus maximus, and vastus lateralis are tagged, and the prosthesis is cemented in place; (**C**,**D**) Muscular cuff is tensioned securely before being sutured to the mesh.

All patients underwent resection of the proximal femur without preservation of the greater trochanter even if the preoperative imaging showed that it could be preserved based on the tumor location. A 10 × 14-inch sheet of sterile Marlex mesh (Bard, Franklin Lake, NJ, USA) was soaked in betadine and then wrapped circumferentially around the proximal aspect of the implant stem and secured to the implant with multiple Ethibond sutures (Figure 1A). After the proximal femoral implant was cemented into the femur, the hip was reduced, and any remaining femoral capsule was purse-stringed with a suture around the implant. The surrounding muscles were then appropriately tensioned and repaired directly to the mesh (Figure 1B–D); muscular repair consisted of the abductors, short external rotators, vastus lateralis and gluteus maximus. Post-operatively, patients wore an abduction brace set to 15° of abduction and 0–70° of hip flexion for 3 months. Patients were instructed to be 50% partial weight-bearing for 6 weeks and then advance to 100% weight bearing over the next 6-weeks. At 3-months post-operative the abduction brace was discontinued and abductor strengthening was allowed.

### 2.2. Primary and Secondary Outcomes

The electronic medical records of all included patients were reviewed to assess post-operative outcomes. The primary outcomes were the ability to ambulate, gait aid utilization, and the presence of a Trendelenburg limp. This was determined based on clinical notes at 3 months, 6 months, and final follow-up. Secondary outcomes were clinical outcome scores, including the MSTS and Harris Hip Scores [13,14], survival, and complications. Statistical analysis was limited to descriptive metrics as there was no comparator group. Survival was assessed using the Kaplan–Meier method.

## 3. Results

### 3.1. Ambulatory Status

All patients were ambulatory throughout the follow-up duration. At 3 months post-op, 17% of patients walked outside of the home without gait aids, 40% used a single cane or crutch, and 50% required a walker or two crutches. At 6 months, 28% used no gait aids, 61% used a single-arm gait aid, and 11% required a double-arm gait aid. At the final follow-up, 44% of patients were ambulatory without assistive devices, and 44% of all these patients had a Trendelenburg gait pattern. Of the eight patients who were ambulatory without gait aids at the final follow-up, six (75%) had a non-Trendelenburg gait pattern.

### 3.2. Clinical Outcomes

The mean MSTS score at the final follow-up was 23 (range 13–30) out of a possible 30 points. The mean Harris Hip Score was 79% (range 66–98) at 6 months. Two patients who underwent PFR for metastatic disease developed new lesions near the implant stem, which were treated with radiotherapy. Overall survival was 94% at 6 months, 89% at 12 months, and 71% at 24 months. The cause of death in all non-surviving patients was their underlying malignancy (renal cell carcinoma, n = 3; osteosarcoma, n = 1).

### 3.3. Complications

There were no instances of post-operative dislocation, loosening, or implant failure. There were no post-operative infections or mesh-related complications. One patient who underwent resection of primary chondrosarcoma of the femur experienced local recurrence and underwent hip disarticulation 12 months after the index procedure.

## 4. Discussion

Reconstruction of the abductor mechanism remains a primary challenge with contemporary proximal femoral replacement (PFR) surgery. Previously, techniques such as trochanteric preservation or direct repair to the implant have been described; however, these strategies are limited in their ability to tension the repair and reattach other muscles of the hip girdle [4,5,6,7,8,9]. The present study used synthetic mesh to reconstruct the peritrochanteric musculature of the proximal femur in patients undergoing proximal femoral replacement for tumor resection or sequelae of metastatic disease. The patients in our series demonstrated high rates of functional ambulation with low rates of mesh-related complications or hip instability.

This study has several limitations. All patients were retrospectively reviewed without a comparator group. As the resections in this study were performed for proximal femoral lesions or sequelae of the oncologic disease, the age range and baseline functional status of patients are broad. Treatment characteristics, including history of radiation and extent of soft tissue resection for primary tumors, varied across patients and added heterogeneity. An additional limitation is the length of follow-up since continued functional improvement or late complications may not have been captured by this study. Given the promising outcomes of this technique, further studies with a larger sample size, a comparator group, and longer follow-up would be beneficial for this subset of patients undergoing proximal femoral replacement.

Ease of ambulation after proximal femoral replacement has been shown to correlate with the strength of the abductor musculature [15]. In the current series with the described technique, all patients were independently ambulatory or used a single-arm gait aid at the final follow-up, and 75% of independently ambulatory patients were able to walk without a Trendelenburg gait. A recent study comparing PFR with trochanteric osteotomy versus soft tissue-only repair found that approximately 50% of patients in both groups required a double-arm gait aid (Table 2) [16]. Another study reported similar results for these two techniques, with overall assistive walking device utilization rates of 68% and 81% for the trochanteric osteotomy and soft tissue-only repair groups, respectively [6]. The rate of non-Trendelenburg gait in the subgroups of the aforementioned studies ranged from 63 to 77%. While the rate of assistive device utilization and Trendelenburg gait in our series is at the lower end of the ranges reported in the literature, differences in technique make direct comparison difficult. Though our results suggest promising outcomes in terms of ambulatory ability and limp-free gait, the mean final MSTS score in our cohort was 76%, which is similar to other abductor reattachment techniques [6]. While others have advocated for the preservation of the greater trochanter when feasible, our institutional experience has been that the trochanter tends to migrate proximally due to tenuous fixation to the implant surface and motion through the repair and implant. Likewise, deliberate removal of the trochanter allows for direct tensioning of the abductor’s tendons to the mesh, potentially enhancing the repair.

Soft tissue deficiency, particularly of the abductors, is a known contributor to instability following proximal femoral replacement [17]. Rizkallah et al. experienced a 19% dislocation rate in patients who underwent PFR with soft tissue-only repair compared to 0% in the trochanteric osteotomy group [6]. Another study reported a 13% dislocation rate across 166 tumor patients undergoing PFR, which included an 11% rate of instability in a subgroup of patients whose soft tissue repair was augmented with an artificial polyester band [18]. Following mesh reconstruction of PFR performed for oncologic indications, we report no dislocations, which suggests that muscular reattachment to the Marlex may provide additional stability to the hip joint.

Furthermore, there were no mesh-related complications in our series. A 1998 study describing capsular reconstruction with mesh and PFR noted that two patients tore through Marlex mesh after late dislocations, and a third patient had no identifiable Dexon mesh upon reoperation for recurrent dislocations [19]. Infection is a primary concern regarding the use of synthetic mesh in hernia repair, and reported infection rates of non-Gore-Tex mesh vary from 2 to 10% [20,21]. This should certainly be a consideration when using mesh around megaprostheses, given the potentially devastating consequences of infected implants; however, none of the patients in our series experienced a superficial or deep wound infection. Other facets of perioperative care, including antibiotic cement and negative-pressure dressings, may contribute to low rates of infection, though the absence of any infection suggests Marlex mesh use may be better tolerated in the hip.

Medical use of synthetic mesh was popularized in the 1960s as a reliable way to reinforce hernia repair [10]. Products such as Dacron and Marlex mesh are flexible yet have high tensile strength, making them ideal for the repair of soft tissue defects; however, enthusiasm for these biomaterials has faded for general surgeons as they produce local fibrosis resulting in the formation of adhesions [10,11,12]. While scarring is undesirable for intraabdominal mesh placement, fibroblastic change at a tendon–mesh interface may allow for stronger reconstruction in instances of tendon detachment, such as proximal femoral replacement. The one patient in our series who underwent reoperation did so at 11 months from PFR for local recurrence of chondrosarcoma. Pathologist evaluation of the proximal femur specimen revealed a surrounding fibrous scar that was firmly adherent to the synthetic mesh material overlying the surface of the prosthesis.

## 5. Conclusions

Hip instability and abductor deficiency following proximal femoral replacement remain challenges. Mesh augmentation of PFRs allowed for adequate soft tissue tensioning and muscular attachment to the body of the implant. All patients undergoing this novel technique were ambulatory without gait aids or with a single-arm assistive device after 3 months post-operative, and 33% of all patients achieved an independent, non-Trendelenburg gait pattern. In our series, this technique was durable, with no dislocations and no mesh-related complications. In summary, mesh augmentation of PFRs may be considered during reconstruction for oncologic indications.

## Figures and Tables

**Table 1:** Patients Undergoing Proximal Femur Replacement.

Patient	Age	Sex	Primary Diagnosis	Pathologic Fracture	Post-Operative MSTS	Post-Operative Gait Aid	Alive/Dead	Duration Follow-Up (Months)
1	63	Male	Renal Carcinoma	Yes	90	Cane	Dead	6
2	59	Male	Renal Carcinoma	No	67	Cane	Dead	10
3	39	Male	Renal Carcinoma	No	93	None	Alive	12
4	62	Female	UPS	Yes	57	Cane	Alive	13
5	72	Male	Renal Carcinoma	No	93	None	Alive	19
6	77	Female	Renal Carcinoma	No	63	Cane Walker for Distances	Alive	20
7	37	Female	Osteosarcoma	No	60	Cane	Dead	22
8	58	Female	Renal Carcinoma	No	77	Single Crutch	Alive	22
9	66	Male	Renal Carcinoma	No	93	None	Dead	23
10	68	Male	Prostate Carcinoma	Yes	100	None	Alive	24
11	70	Male	Radiation Associated Fracture	No	87	None	Alive	24
12	60	Male	Mast Cell Sarcoma	Yes	93	None	Alive	28
13	66	Male	Renal Carcinoma	No	100	None	Alive	30
14	46	Female	Chondrosarcoma	No	43	Single Crutch	Alive ^1^	33
15	70	Female	Breast Carcinoma	Yes	67	Cane	Alive	34
16	78	Female	Renal Carcinoma	Yes	57	Cane	Alive	39
17	62	Female	Lung Adenocarcinoma	Yes	80	None	Alive	43
18	75	Female	Renal Carcinoma	Yes	63	Cane	Alive	44

MSTS: Musculoskeletal Tumor Society Score; UPS: Undifferentiated Pleomorphic Sarcoma. ^1^ Patient number 14 underwent hip disarticulation for local recurrence.

**Table 2:** Comparison of Abductor Repair Techniques.

Study	Abductor Repair Method	Group Size	Ambulatory	Ambulatory without Gait Aid	Non-Trendelenburg Gait	Infection	Dislocation
Groundland et al. [16]	Trochanter preservation	n = 29	90%	17%	10%	17%	0%
	Soft tissue repair to prosthesis	n = 24	100%	33%	38%	4%	4%
Rizkallah et al. [6]	Trochanter preservation	n = 22	NR	32%	23%	9%	0%
	Soft tissue repair to prosthesis	n = 31	NR	19%	26%	10%	19%
Broida et al. (Present)	Mesh-augmented soft tissue repair	n = 18	100%	56%	33%	0%	0%

## Data Availability

The raw data supporting the conclusions of this article will be made available by the authors on request.
